# Effect of Liquid Crystalline Host on Structural Changes in Magnetosomes Based Ferronematics

**DOI:** 10.3390/nano11102643

**Published:** 2021-10-08

**Authors:** Peter Bury, Marek Veveričík, František Černobila, Matúš Molčan, Katarína Zakuťanská, Peter Kopčanský, Milan Timko

**Affiliations:** 1 Theoretical Department of Physics, FEIT, Žilina University, Univerzitná 1, 010 26 Žilina, Slovakia; vevericik@fyzika.uniza.sk (M.V.); cernobila@fyzika.uniza.sk (F.Č.); 2 Institute of Experimental Physics, SAS, Watsonova 47, 040 01 Košice, Slovakia; molcan@saske.sk (M.M.); zakutanska@saske.sk (K.Z.); kopcan@saske.sk (P.K.); timko@saske.sk (M.T.)

**Keywords:** liquid crystals, ferronematics, magnetosome particles, SAW attenuation response

## Abstract

The effect of the liquid crystalline host on structural changes in magnetosomes based on ferronematics is studied using the surface acoustic wave (SAW) technique supported by some capacitance and light transmission measurements. The measurement of the attenuation response of SAW propagating along the interface between LC and the piezoelectric substrate is used to study processes of structural changes under magnetic field. The magnetosome nanoparticles of the same volume concentration were added to three different nematic LCs, 5CB, 6CB, and E7. Unlike to undoped LCs, the different responses of SAW attenuation under the influence of magnetic and electric fields in LCs doped with magnetosomes were observed due to characteristic structural changes. The decrease of the threshold field for doped LCs as compared with pure LCs and slight effects on structural changes were registered. The threshold magnetic fields of LCs and composites were determined from capacitance measurements, and the slight shift to lower values was registered for doped LCs. The shift of nematic-isotropic transition was registered from dependencies of SAW attenuation on temperature. The acoustic anisotropy measurement approved the previous supposition about the role of bulk viscosity in used SAW measurements. In addition, capacitance and light transmition investigations supported SAW results and pointed out conclusions about their magnetic field behavior. Obtained results are discussed and confronted with previous ones and coincide well with those observed using acoustic, optical, or dielectric techniques.

## 1. Introduction

The improvements in nanomaterial preparation and control of properties for nanometre-scale structures during the last decades enable the development of new products, applications, and technologies in high-tech devices and in medicine [1,2]. From the designing point of view, liquid crystal-based nanomaterials are very attractive as they can be aligned or reoriented by external stimuli such as temperature, light, magnetic, and/or electric fields. Besides, combining the unique properties of nanoparticles with the anisotropy of liquid crystals (LCs) creates new opportunities for tuning the properties of LCs for novel materials that contribute to the nanotechnology revolution, mainly in areas such as nanoscale electronics, electro-optics, sensors and display devices [3,4,5]. Nematic LCs doped with magnetic nanoparticles (ferronematics) are magnetically active anisotropic fluids, which are a manifestation of the original idea [6], suggesting that doping liquid crystals by fine magnetic particles may enhance their sensitivity to magnetic fields. The theoretical models concerning the LC-magnetic particles interaction and their behavior in magnetic field [7,8,9] and following experimental investigation on ferronematics [10,11,12] confirmed that magnetic particles are essentially affected by external fields and verified that the interaction between the LC molecules and magnetic particles is interesting in term of practical application and entirely complicated. The basic pecularity of ferronematics is a coupling between magnetic moments and the nematic LC matrix. The coupling between average magnetic moment ***m*** and director ***n*** characterizing the preferential direction of the nematic molecules under applied magnetic field can result in the reorientation of LC molecules [13,14]. The small volume concentration of magnetic particles can ensure that the inter-particle magnetic dipole-dipole interaction can be ignored.

For more then last two decades, magnetosomes have attracted scientific interest due to their interesting biophysical properties and potential applications [15,16]. Magnetosomes are magnetotactic bacteria that belong to a phylogenetically and morphologically diverse group of microorganisms that can align in and navigate along geomagnetic fields. Recent studies have also shown that magnetosomes possess a high efficacy in hyperthermia treatment [17,18]. Magnetosomes are synthesized in a biomineralization process by a group of bacteria called magnetotactic bacteria. They consist of a nanometer-sized magnetosome crystal (magnetite Fe_3_O_4_ or greigite Fe_3_S_4_) and a bilayer membrane surrounding the crystal [19]. They are magnetic monodomain, well-crystallized, and thermally stable nanoparticles at the physiological temperature with a limited size distribution [15,20,21]. Magnetosomes can also form linear chains and eventually closed loops [22]. The magnetic properties of magnetosomes depend on the crystal purity and chain formations, including crystal size and their arrangwment along the magnetic axis [23,24].

One useful tool for investigating structural changes in LCs, especially those doped with various kinds of nanoparticles, appears technique using the surface acoustic wave (SAW) [25,26,27]. Comparing with the case when the longitudinal wave is used, the SAW technique has advantage because only a very thin layer of LC is required for the experimental investigation. The SAW generated on a piezoelectric substrate propagates along the surface as an elliptically polarized wave as long as SAW arrives the LC located on its way. The SAW amplitude, due to the generation of a longitudinal wave into LC, is then damped and SAW propagates at the LC/substrate interface further as a leaky SAW. The generation of a longitudinal wave into LC is caused just by the vertical displacement of original SAW at the surface. Two different waves are then propagating in this way, represented particularly by longitudinal and shear vertical particle displacement. As the longitudinal wave is strongly absorbed by LC, which is subsequently indicated by changing SAW amplitude in the direction of SAW propagation, it can indicate any changes in LC structure. Hence, SAW technique can be applicable for utilization in the study of LC structural changes since a SAW attenuation response reflects any changes in the orientation of LC molecules that result from field application or varied external conditions.

In some previous works [27,28,29] the effect of various types of magnetic nanoparticles with respect to their shape, i.e., nearly spherical, chains of magnetosomes from magnetotactical bacteria and rod like nanoparticles, on the behavior of nematic LCs in magnetic field, including magnetic Freedericksz transition and light transmission was studied. It was shown that the shape and size of nanoparticles has significant influence on the interaction between particles and molecules of LC and by that on structural transitions. In this paper we present for the first time the results of investigation of influence of chains of magnetosomes of the selected size and concentration as magnetic particles in several different LCs (5CB, 6CB, and E7) to determine their effect on structural changes mainly under external magnetic but also electric fields as well as the effect on the shift of threshold fields and transition temperatures for individual LCs are presented. The SAW amplitude response was used as a main investigating technique, supported by optical (light transmission) and capacitance measurements.

## 2. Experimental Details

### 2.1. Magnetosomes

Investigated magnetosomes were magnetic crystals of iron oxide nanoparticles (magnetite Fe_3_O_4_) covered and connected by a biological membrane (phospholipid bilayer) and formed chains. The chain of magnetosomes can consist of 12–20 crystals, which multiplies their sensitivity to the magnetic field. Magnetosomes were cultivated and extracted from the Magnetotacticum Spirillum AMB 1 bacteria in laboratory conditions using the biomineralization process already described as well as their other properties [28,30,31]. The isolation process of magnetosome chains from the body of the bacteria addressed one general recipe and three consecutive cycles with different buffers [30].

**Figure 1:**
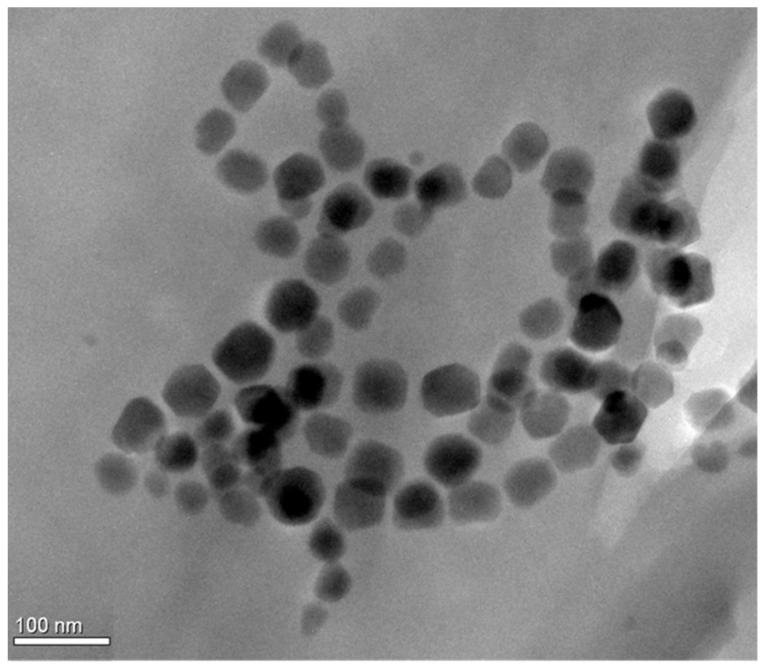
TEM image of isolated magnetosomes.

The TEM analysis was performed in a Philips CM300ST FEG TEM, and the image in Figure 1 represents a few long chains of isolated magnetosomes.

**Figure 2:**
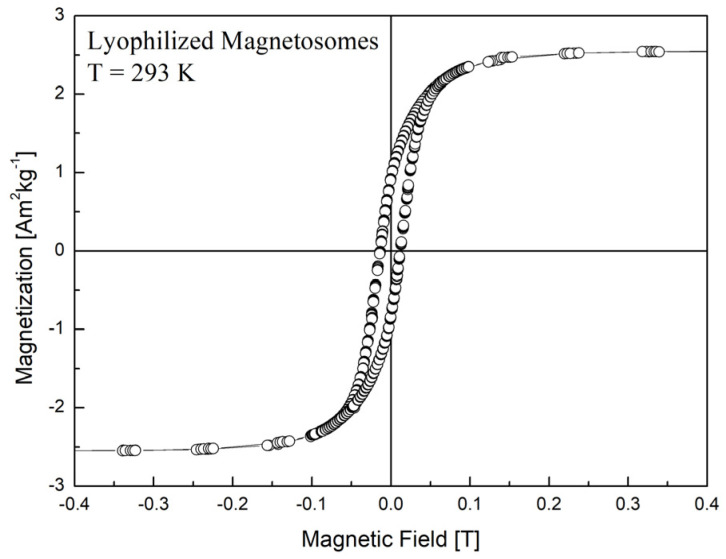
Magnetization curve of isolated magnetosomes in the powder form.

Chains composed of several particles are clearly visible. The size distribution of magnetosome particles that was studied using the procedure published previously [32], led to the following mean size: 43 ± 12 nm. Magnetization measurements of the prepared isolated magnetosomes in the powder form were carried out by a Vibrating Sample Magnetometer installed on a cryogen-free superconducting magnet (Cryogenic Ltd., London, UK). The magnetization curves (Figure 2) were measured at room temperature and an applied magnetic field up to 0.4 T. 

### 2.2. LC Sample Preparation for Experiments

For the experiments, liquid crystals 4-cyano-4′-alkylbiphenyls with alkyl group pentyl (5CB) and hexyl (6CB) and E7, which is a mixture of four liquid crystals-5CB, 4-cyano-4′-n-heptyl-biphenyl (7CB), 4-cyano-4′-n-octyloxy-biphenyl (8OCB) and 4-cyano-4″-n-pentyl-terphenyl (5CT), were chosen. The composites of liquid crystal and magnetosomes with the concentration of 0.2 wt% were prepared by the following procedure. Magnetosomes of required mass in powder form were dispersed in cyclohexane, and to break clusters, the mixture was sonicated for 10 min and afterward stirred for 16 h at room temperature. After that, the liquid crystal was admixed, and subsequently, the sample was stirred in the isotropic phase for 16 h to allow the cyclohexane to evaporate.

### 2.3. Capacitance Measurements

The LC cells consisting of two glass plates with transparent ITO electrodes and rubbed polyimide layer that ensures the planar alignment of liquid crystal molecules were filled with the sample for the capacitance measurements. Before the filling, the composites were sonicated in the isotropic phase. To determine the isotropic to nematic transition temperatures, the dependences of capacitance on temperature were obtained from measurements performed on samples in cells with thickness *D* = 5 µm. Afterward, the magnetic dependences of capacitance were acquired at the temperature of 25 °C for samples in cells with *D* = 50 µm and *D* = 20 µm, respectively. The capacitance was measured at the frequency of 1 kHz by a high-precision capacitance bridge (Andeen Hagerling). To compare the data for pure liquid crystals and liquid crystals doped with magnetosomes, dependences of reduced capacitance (*C* − *C_max_*)/(*C_max_* − *_min_*) on temperature and magnetic field were plotted. *C* denotes actual capacitance where *C_max_* and *C_min_* are values of capacitance measured at a maximal and minimal value of temperature and magnetic field.

### 2.4. SAW Measurements

The LC cell of the thickness 100 μm was prepared right on the center of the LiNbO_3_ substrate, supplied by two interdigital transducers. The LC layer was thus sandwiched between the delay line and the optical glass plate, both coated with gold electrodes. The thickness of LC layer was provided by adequate spacer. The substrate with LC cell was installed in the sample holder inserted in the measuring chamber. SAW pulses of frequency 10 MHz and wideness of ~1 µs were generated by the first interdigital transducer using high frequency pulses of the Pulse Modulator and second transducer received SAW signal passing through the interface between substrate and LC cell that was processed in the following by the Receiver (MATEC 7700, USA). The response of SAW attenuation *α* was recorded by MATEC Attenuation Recorder 2470 A. To register shorter times when the jumped fields were applied, the Gated Integrator and Boxcar Averager SR 250 were used. Programable 15 Hz Function Generator HM 8131-2 was used as DC-supply. The configuration of experimental arrangement has already been described [25,27]. The maximal determined deviation for the measurement of attenuation changes Δ*α* was ±0.02 dB and for magnetic field determination was 0.1–1.0 mT, depending on the magnetic field magnitude. The temperature could be stabilized in the range of 5–80 °C with an accuracy of ±0.1 °C. 

### 2.5. Light Transmission Measurements

To find the effect of magnetosomes on LC behavior, light transmission investigations were done in similar LC cells as in the case of capacitance measurements (*D* = 50 μm), that is they consist of two float glasses coated with ITO transparent conductive layers, and alignment layers rubbed in a parallel direction to electrodes to promote the parallel alignment. The Green DPPS Laser Module CW532 (Roithner LaserTechnik GmbH, DE) generated laser beam (532 nm, 5 mW) illuminated the cell’s glass in normal incidence using an optical triangular prism and polarizer. The initial position of this polarizer, for all investigated suspensions, ensured the linearly polarized incident light beam, and the position of LC cells provided for maximal transmittance registered by a photodetector. The second polarizer was adjusted peralelly with first one or could be also crossed. After passing another polarizer and prism, the intensity of transmitted light was recorded by a photodetector (Thor LABS PDA36A 350–1100 nm) connected to the multimeter and subsequently registered by a computer monitoring the light intensity as a function of magnetic or electric field, alternatively time. The scheme of experimental configuration has already been described [26,29]. The light transmission was expressed in the case of parallel polarizers as *I*/*I*_0_, here *I*_0_ and *I* are the intensity of incident light passing through the LC cell without applied field and under the field, respectively. In the case of crossed polarizers the light transmission was expressed as (*I* − *I*_0_)/*I*_0_.

## 3. Results and Discussion

### 3.1. Capacitance Measurements

**Figure 3:**
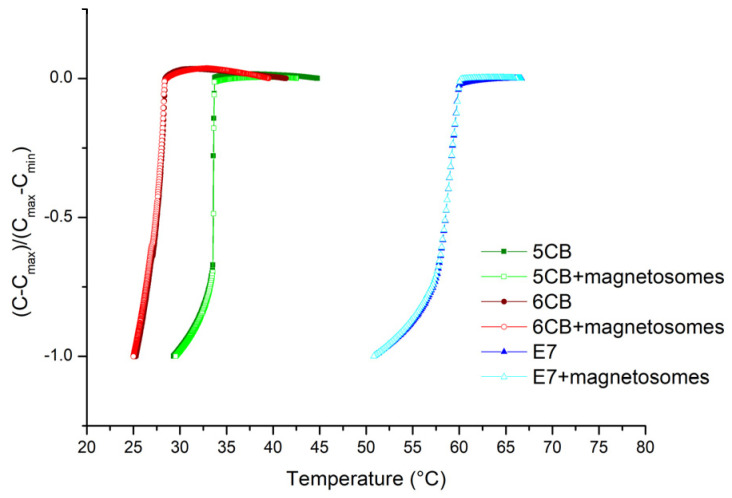
Temperature dependence of reduced capacitance of the pure LCs (5CB, 6CB, E7) as well as doped with magnetosome particles of concentration 0.2 wt%.

Measured temperature dependences of reduced capacitance illustrated in Figure 3 shows that curves for composites lie basically on the curves for pure liquid crystals.

**Figure 4:**
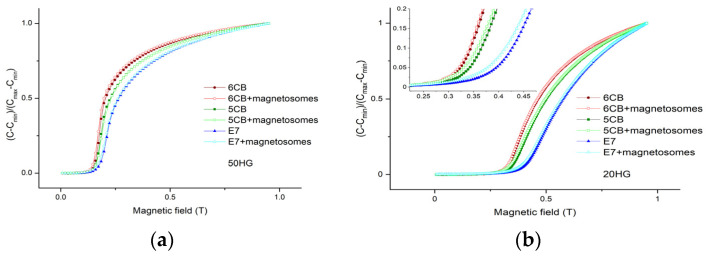
Reduced capacitance dependence of the pure LCs (5CB, 6CB, E7) as well as doped with magnetosome particles of concentration 0.2 wt% on the external magnetic field for samples in 50 µm (**a**) and 20 µm (**b**) cells. The insert in (**b**) shows detail in the vicinity of threshold magnetic fields.

From the measurements the isotropic to nematic phase transition temperatures were determined as 33.7 °C, 28.4 °C, and 60.0 °C for 5CB and 5CB with magnetosomes, 6CB and 6CB with magnetosomes, E7 and E7 with magnetosomes, respectively. Figure 4 shows the dependences of reduced capacitance on magnetic field for samples in 50 µm cells (a) and 20 µm cells (b), respectively. 

Since the threshold magnetic field depends inversely proportional on the sample thickness, the measurements in 50 µm show cell full transition region while the measurements for samples in 20 µm cell show the region around threshold magnetic field. The reduced capacitance dependences on the magnetic field acquired for samples in 20 µm cells (Figure 4b) show a slight shift to lower values for liquid crystal 5CB and E7 doped with magnetosomes compared to pure liquid crystals. The threshold magnetic fields of liquid crystals and composites in 20 µm cells are listed in Table 1.

### 3.2. SAW Measurements 

The initial intrinsic arrangement of LC in the case of the SAW attenuation measurement was supposed to have a predominate alignment in the plane of electrodes (director ***n*** parallel to electrodes), and the magnetic field was then applied perpendicular to them. The applied magnetic field should turn LC molecules due to the coupling between magnetic moments of magnetosomes and LC molecules, starting at the center of the LC cell, to the perpendicular direction with respect to the electrodes surface. When an LC is placed on the SAW path on the piezoelectric substrate, the SAW amplitude after reaching an LC layer is exponentially attenuated because when the substrate is in contact with LC, SAW radiates a longitudinal wave into LC under Rayleigh angle *θ_R_*, giving rise to the propagation losses [33]. Due to the strongly absorbed longitudinal wave generated by SAW into the LC, the SAW attenuation *α* subsequently responds to any changes in LC orientational structural changes initiated by external conditions (temperature, magnetic and electric fields, illumination). The total attenuation α through that path along the substrate/LC interface is then the sum of the attenuation of a fluid-damped Rayleigh SAW α*_s_* and the attenuation of a plane longitudinal wave *α_lc_* in a viscous LC. As α*_s_* is practically independent of viscosity coefficients$,\;\alpha_{s}=\rho_{lc}v_{lc}/\left(\rho_{s}v_{s}\lambda_{SAW}\right)$, measured SAW attenuation changes are registered due to the changes in the attenuation of a longitudinal wave *α_lc_* propagating into a liquid crystal. Here $v_{lc}$ is the velocity of the longitudinal wave in LC and *ν_s_* is the velocity of the Rayleigh wave on the substrate, symbols *ρ_lc_* and *ρ_s_* are the LC and substrate densities, respectively, and *λ_SAW_* is the SAW wavelength. The comparison of experimental results with the theory using hydrodynamic approximation [27,34,35,36] uniquely determined that the bulk viscosity should dominate in the attenuation in the present SAW technique.

**Figure 5:**
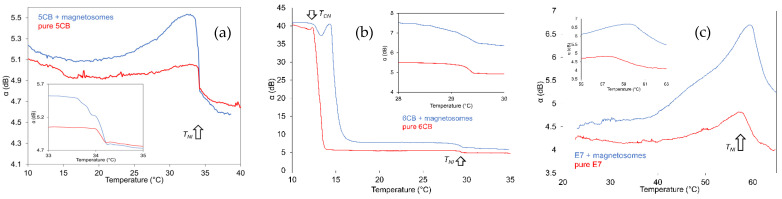
Comparing of temperature dependences of SAW attenuation measured for 5CB (**a**) 6CB (**b**) and E7 (**c**) doped with magnetosomes of concentrations 0.2 wt% with that of corresponding pure LCs.

The dependences of SAW attenuation on temperature measured for LCs 5CB, 6CB, and E7 containing magnetosome nanoparticles of the same concentration 0.2 wt% are illustrated in Figure 5a,c.

The temperature development of the SAW attenuation indicates the different role of thermal motion on the structural changes detected through SAW interaction with different types of LC molecules. Presented temperature dependences also show the effect of magnetosomes on the shift of nematic-isotropic transition temperature *T_NI_* for individual doped LC types compared to the corresponding pure ones. The most evident shift of the temperature *T_NI_* corresponding to structural changes to the isotropic phase (*T_NI_*) was registered in the case of E7 (~2.3 °C) toward higher temperatures. However, in the case of 5CB and 6CB the shift of temperature *T_NI_* toward lower temperatures was registered, ~1.4 °C and ~1.1 °C, respectively.

Interestingly, for latter referred LCs (5CB and 6CB), the *T_NI_* shift is succeeded by the slower transition to the isotropic state. The shift of phase transition towards lower temperatures for doped LC can be attributed to the anchoring interactions between LC molecules and nanoparticle surfaces that disturb the LC order. This effect predominates the effect concerning the permanent polarization of nanoparticles that assists in the increase of phase transition temperature [37]. Another reason for nematic-isotropic transition decrease is related to the increasing volume of impurities occurring in LCs during their synthesis due to the increased nanoparticles concentration [38]. However, the shift of the transition point towards higher temperature, as in E7, could be attributed also to magnetosomes that induce local magnetic moments with a sufficient number of neighboring LC molecules. The addition interaction can then lead to an increasing transition point. The behavior of LCs at temperature in the vicinity of the transition point can be generally explained by increase in the bulk viscosity coefficients or the fluctuation of order parameter [29,39,40]. Concerning the total registered changes of the SAW attenuation corresponding to structural changes in the vicinity of transition points *T_NI_*, which are evident in detail inserts of Figure 5, they are very similar for all investigated LCs at the used frequency 10 MHz (Δ*α* ~1.2–1.5 dB). 

While in the cases of 5CB and E7 (Figure 5a,c), no crystal-nematic transition (*T_CN_*) was registered, the SAW attenuation in the case of 6CB (Figure 5b) shows at the temperature corresponding to the crystal-nematic transition sharp decrease followed by the stable attenuation with increasing temperature up to the temperature of ~30 °C when the structural changes to isotropic phase (*T_NI_*) are represented by a weak decrease of the SAW attenuation (see also detail in the insert). However, comparing this transition for pure and doped 6CB, following the attenuation decrease at the transition temperature *T_CN_* another peak was observed at temperatures ~14.5 °C. This peak, observed also previously in 6CB doped with suprionic nanoparticles [26], can be attributed to the polarization process connected with the double layer creation on electrodes. 

Comparing SAW results (Figure 5) with capacitance measurements (Figure 3), where no evident shift of the transition point due to the doping process was registered, another evident difference could be seen. Except for the nonregistered shift of the transition points in the cases of doped LCs in capacitance measurements, practically stable capacitance in the isotropic state could be observed in capacitance measurements for all investigated LCs. However, in the SAW attenuation, only 6CB shows such behavior. 5CB and especially E7 shows the gradual transition to isotropic phase and on the side also the active influence of magnetic field in the temperature region above *T_NI_*. 

**Figure 6:**
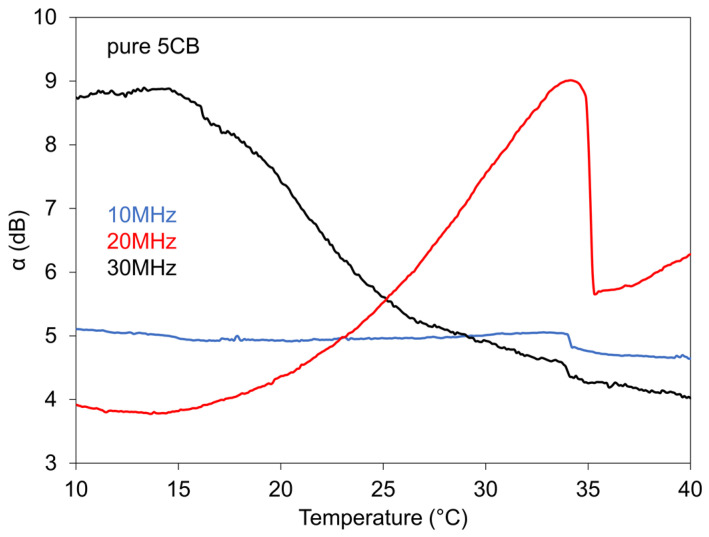
Dependences of SAW attenuation on temperature for pure 5CB measured at different frequencies.

The influence of used frequency on measured dependences of SAW attenuation on temperature for pure 5CB, as a representative sample, can be seen in Figure 6 for three various frequencies (10 MHz, 20 MHz, and 30 MHz). 

It stands to reason that SAW of different frequencies is differently sensitive to processes occuring in LCs during varied temperature. The measurement at higher frequency (40 MHz) could not distinguish any transition point. Similarly, marked frequency effect on acoustic attenuation was also registered in the case of another investigated LCs. If we take account of the supposition that at the frequencies region that corresponds to realized SAW measurements the bulk viscosity coeficients are frequency-dependent in contrast with the shear viscosity coefficients that are frequency-dependent at high frequencies and [27,41,42], then observed temperature dependences again support the supposition about the responsibility of bulk viscosity coefficients for used SAW results, similarly as in the cases when longitudinal acoustic waves were used. 

**Figure 7:**
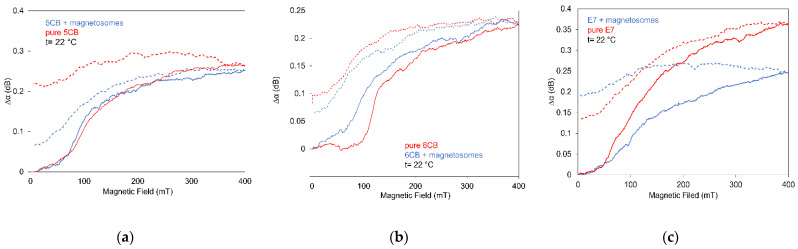
Effect of magnetic field on SAW attenuation for 5CB (**a**) 6CB (**b**) and E7 (**c**) doped with magnetosomes of concentrations 0.2 wt% with that of corresponding pure LCs for increasing (full lines) and decreasing (dushed lines) magnetic field.

SAW attenuation dependences on linearly increasing magnetic fields ***B***, perpendicular to the cell and wave vector ***k****_SAW_*, at temperature 22 °C and constant rate (15 mT/min) measured for LCs doped with magnetosomes are illustrated in Figure 7 (full curves). This Figure presents SAW attenuation changes for samples based on 5CB (a), 6CB (b), and E7 (c) doped with magnetosomes of the same concentrations 0.2 wt%, including corresponding pure LCs. Furthermore, it shows the effect of magnetosome nanoparticles on the decrease of the threshold field regarding pure LCs and the role of magnetosomes on structural changes. All observed dependencies, similarly as in the case of many other ferronematics, can be resolved into three main phases, very weak increase depending on the type of LC, in investigated samples up to ~50–70 mT, followed by a faster increase which gradually merges into saturation. The main reorientation processes of LC molecules are realized during the faster increase of the attenuation. Therefore, it was very important to find that the effect of magnetosomes on LC compounds behavior under magnetic field was very weak (5CB, 6CB), or even doping gave rise to the lowering of the field-effect (E7).

Concerning the behavior observed in the decreasing regime (memory effect), in all cases of investigated samples (dotted curves), except the hysteretic development, the significant remainder attenuation was registered at zero magnetic field, including pure LCs. Similar behavior of dependences Δ*α* on magnetic field ***B*** was observed for 6CB but doped with spindle magnetic particles [43]. However, comparing the presented results with those obtained on 6CHBT liquid crystal, doped with chain-like, rod-like, spherical or nanorod magnetic nanoparticles, eventually with carbon nanotubes, the residual behavior belongs here to more marked. However, the development in decreasing magnetic field and by that the residual structural changes registered by SAW attenuation can be influenced, besides the kind of LC, also by the length and shape of magnetosome chains. It should be noted that under jumped change of magnetic field, induced changes after magnetic field removal vanished in a tenth of seconds.

**Figure 8:**
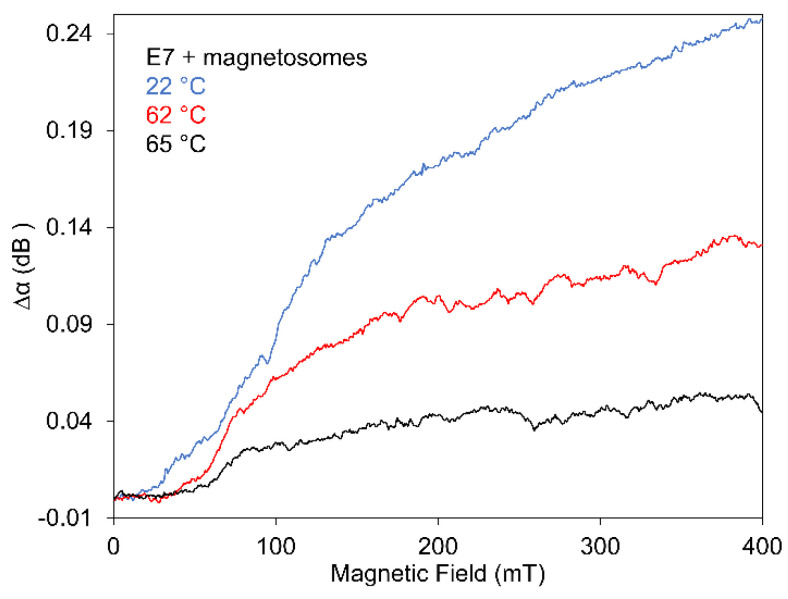
Comparing of the effect of magnetic field on SAW attenuation for E7 doped with magnetosomes of concentrations of 0.2 wt% for three temperatures around *T_NI_* (60 °C).

The dependences of SAW attenuation on the magnetic field for three different temperatures near the transition point of doped E7 (~60 °C) are illustrated in Figure 8. This Figure presents the decreasing SAW attenuation changes with temperature using three dependences measured from room temperature (22 °C) through the transition point up to isotropic state (65 °C). However, it also shows that structural changes are occurring even at temperatures higher than *T_NI_*. This fact could be affected by LC molecules in magnetosomes surrounding.

The character of the effect of magnetosomes on the development of the SAW attenuation under applied electric field in investigated LC samples corresponds in principle to the development under magnetic field, reflecting the corresponding structural changes. The voltage linearly raised from zero to 10 V in 10 min. The measurement of SAW attenuation registered a slight decrease of the threshold voltage and, except 5CB, also the lowering of the field-effect on the structural changes. The observed development of SAW attenuation consisted of three regions, similarly as in the case of magnetic field or as in the case of LC compounds doped with ferroelectrics [44,45]. Obtained results demonstrate that the effect of the electric field that can initiate the reorientation of the director towards its direction is different for investigated LCs. While in the case of 5CB, doping with magnetosomes causes larger structural changes and only slight differences in the development compared to pure LC, in the case of both 6CB and E7, the effect of the electric field consists of the decreasing effect of the electric field regarding the pure LCs, and in the case of 6CB even more noticeable. So that the markedly larger and positive change of acoustic attenuation compared to pure LC and the larger effect of doping with magnetosome nanoparticles is evidently detected only in the case of 5CB.

**Figure 9:**
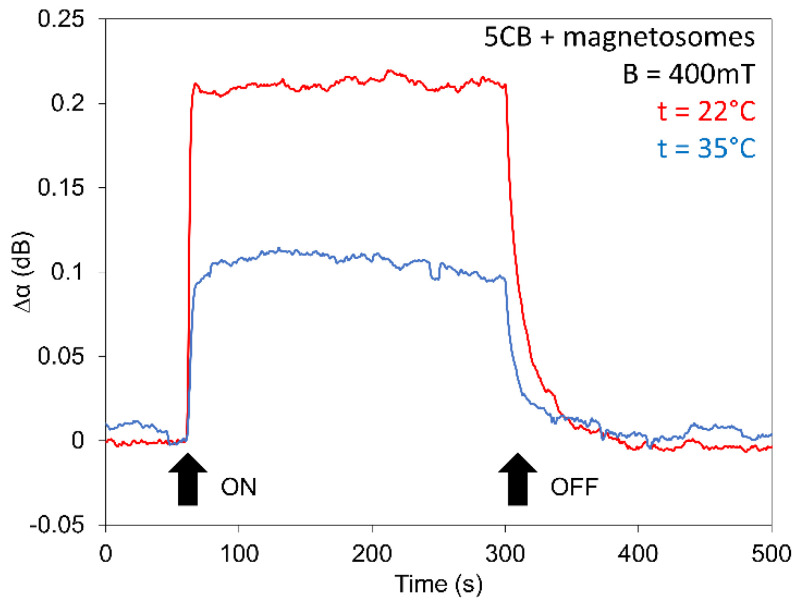
Effect of jumped magnetic field (400 mT) on SAW attenuation of 5CB doped with magnetosomes (0.2 wt%) at two different temperatures, one in nematic (22 °C) and another in isotropic (35 °C) state.

**Figure 10:**
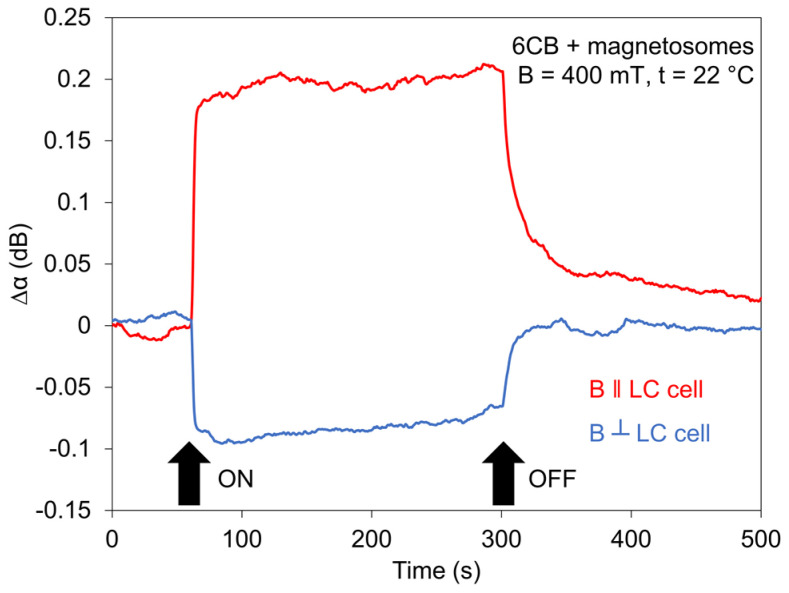
Effect of jumped magnetic field (400 mT) on SAW attenuation of 6CB doped with magnetosomes (0.2 wt%) at two different magnetic field orientations, ***B*** ⊥ LC cell, and ***B*** ‖ LC cell.

**Figure 11:**
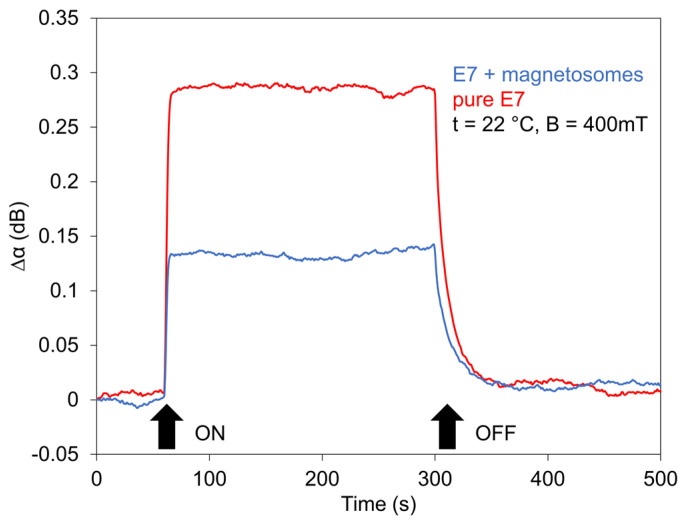
Effect of jumped magnetic field (400 mT) on SAW attenuation of E7 doped with magnetosomes (0.2 wt%) compared with pure LC.

The development of the switching processes for all investigated LCs for jumped magnetic field (400 mT), in addition, measured for different temperatures or different field orientation, is shown in Figure 9, Figure 10 and Figure 11. The magnetic field in the jumped regime fas been applied during 120 s. Experimental results confirmed on one side previous results that structural changes initiated by jumped magnetic field reached the saturated state depending upon the intensity of magnetic field [25] and on another side, they pointed out practically to the independence of the rise time of doping by magnetosomes.

The same situation was in the case of the decreasing regime (decay time), where the magnetosomes played an inessential role. Furthermore, they pointed out that LCs, after passing across the transition point *T_NI_*, remain magnetically active. The results obtained for two different orientations of the magnetic field, magnetic field ***B*** perpendicular to both the cell plane and ***k_SAW_*** and magnetic field ***B*** laying in the cell plane but perpendicular to ***k_SAW_*** (Figure 10), also shows different (opposite) behavior of SAW attenuation due to the different correlative orientation of ***B***, ***k_SAW_***, and ***n***. Similar results were observed in the SAW study of 6CB doped with spindle-like magnetic particles which have magnetic moments oriented perpendicularly to their long axes [43] or in nematic LCs doped with magnetic needles, studying the birefringence [45].

**Figure 12:**
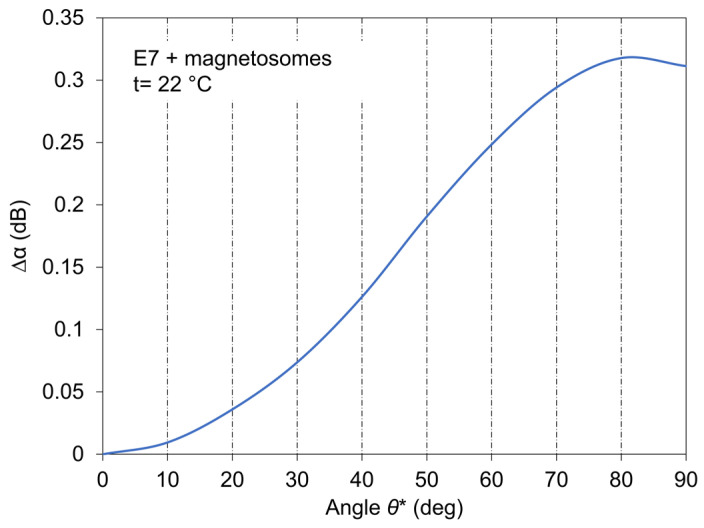
Angle dependence (anisotropy) of SAW attenuation measured at ***B*** = 300 mT for E7 doped with magnetosomes.

The angle dependence of SAW attenuation, Δ*α* (θ*), for E7 doped with magnetosomes measured at magnetic field 300 mT is shown in Figure 12 as a representative measurement.

The angle θ* is here the angle between magnetic field orientation and direction perpendicular to the Plane of LC cell. As the SAW attenuation consists of surface and volume attenuation, *α_s_* and *α_lc_*, respectively, and the surface attenuation *α_s_* is practically constant in our measurements (calculation gives value ~175 m^−1^), the measured SAW attenuation, which increased with increasing angle should be caused by the volume component of attenuation *α_lc_*. The angle was changed by 10 degrees after 2 min of stabilization from perpendicular orientated magnetic field ***B*** (0 degrees) to parallel orientation (90 degrees). The presented dependence is then the fitting of measured points. The relation between measured angle θ* and angle *θ* related to the direction of a longitudinal wave is given by the expression

(1) $$\theta =\frac{\pi }{2}-\left(\theta^{*}-\theta_{R}\right),$$

where *θ_R_* is the Rayleigh angle. The calculated Rayleigh angle for our arrangement is $\theta_{R}=\sin^{-1}\left(v_{lc}/v_{s}\right)$ ≈ 22 degree. The angle θ* determined from anisotropy measurements is ~10 degrees that is, compared to the calculated value for pure LC, something lower value. However, some reason for the difference can be caused by the fact that longitudinal wave velocity decreases with growing concentration of doped nanoparticles [27,45]. These anisotropy results validates that the bulk viscosity should dominate the attenuation in present SAW investigation. The SAW investigation that is orientated in particular to determination of the role of magnetic dopants on the behavior of LCs under external fields, got to results completely satisfying at acquiring underlying information related to the influence of magnetosome nanoparticles on several LCs behavior. The light transmission measurements are presented for the case when parallel polarizers were used.

### 3.3. Light Transmission Measurements

**Figure 13:**
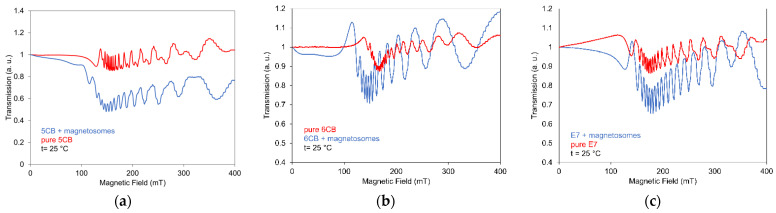
Effect of magnetic field on light transmission for 5CB (**a**) 6CB (**b**) and E7 (**c**) doped with magnetosomes of concentrations 0.2 wt% (**a**) compared with that of corresponding pure LCs.

The magneto-optical behavior was investigated in the same set of LCs including both doped with magnetosome nanoparticles and for comparison pure LCs using the already described arrangement. The dependence of the light transmission on magnetic field mea-sured at increasing regima at a constant rate (15 mT/min) in parallel polarizators arrangement and temperature 25 °C for all investigated LCs doped with magnetosomes, including pure LCs, are illustrated in Figure 13a–c.

This Figure presents the light transmission changes for magnetic field ***B*** perpendicular to the cell plane for individual samples doped with magnetosome particles and is compared with light transmission measured for pure LCs. According to pure LC, magnetosomes caused the marked decrease of the threshold field in 5CB and 6CB and only a slight shift to the higher field in the case of E7. Concerning the role of such magnetic particles on the optical properties, it can be seen that although these particles improved slightly light transmission, however, due to the existing oscillations, by contrast with nanorod, rod-like magnetic nanoparticles or CNTs [25,27,29], they could not be applicable for optical devices. The reason of observed oscillations can be explained by the redistribution of LC molecules around the direction of the nematic director in equilibrium position, after its turns from the planar alignment on the cell surfaces to the perpendicular at the center of the LC layer, when magnetic field increases. Due to the weak influence of magnetosomes’ magnetic moments on LC molecules in all investigated LCs, the oscillations also remain in doped LCs. The light-transmission characteristics without any oscillations, with smooth development, were recently observed in the case of some carbon nanotubes (CNT) [25] and magnetic nanoparticles of rod-like shape [29]. Structural changes were in those cases stable that is important for switching processes application. It is apparent that a decisive role in the stability of magneto-optical behavior that ensures stable magneto-optical responses should play the magnetosome chain-like shape and particularly length of nanoparticle chains as wall as their concentration.

## 4. Conclusions

In this contribution, the SAW technique supported by capacitance and light transmission measurements was used to study the effect of magnetosomes as liquid crystal host on several nematic LCs (5CB, 6CB, and E7) behavior mainly in a magnetic field. The attenuation response of SAW was mainly used to investigate structural changes that take place under magnetic field. The slight decrease of the threshold field for doped LCs according to pure LCs and only slight effects on structural changes were registered. The threshold magnetic fields of LCs and composites were determined from the reduced capacitance dependencies on the magnetic field. The measurement of the temperature dependence of SAW attenuation indicated also the shift of nematic-isotropic transition to lower temperatures in the case of 5CB and 6CB. However, in the case of E7 the shift of nematic-isotropic transition to a higher temperature was registered and that structural changes were occurring even at temperatures higher than *T_NI_* that could be affected by LC molecules in magnetosomes surrounding. The measurement of both the acoustic anisotropy and SAW frequency influence on the α(T) dependence approved our previous assumption about the important role of bulk viscosity in present SAW experiments, typical obviously for cases when longitudinal waves were used. Light-transmission experiments besides supported some SAW results pointed out that particles improved slightly light transmission, however, due to the existing oscillations composities could not be applicable for optical devices. In addition, obtained results were discussed and compared in consideration of previous results and coincide fully with those observed using bothe different magnetic nanoparticles and experimental techniques.

## Figures and Tables

**Table 1:** The threshold magnetic fields of pure liquid crystals and liquid crystals doped with magnetosomes measured in 20 µm cells.

Sample	*Bc* (T)
6CB	0.32
6CB + magnetosomes	0.32
5CB	0.34
5CB + magnetosomes	0.33
E7	0.40
E7 + magnetosomes	0.38

## Data Availability

The data is not available due to further study.
